# Case report: Sevelamer-associated colitis—a cause of pseudotumor formation with colon perforation and life-threatening bleeding

**DOI:** 10.3389/fmed.2023.1097469

**Published:** 2023-04-27

**Authors:** Margareta Fistrek Prlic, Mislav Jelakovic, Marko Brinar, Dora Grgic, Ivan Romic, Zlatko Marusic, Ema Ivandic, Bojan Jelakovic, Ivana Vukovic Brinar, Zeljko Krznaric

**Affiliations:** ^1^Department of Nephrology, Arterial Hypertension, Dialysis and Transplantation, University Hospital Center Zagreb, Zagreb, Croatia; ^2^Department of Gastroenterology, University Hospital Center Zagreb, Zagreb, Croatia; ^3^School of Medicine, University of Zagreb, Zagreb, Croatia; ^4^Department of Abdominal Surgery, University Hospital Center Zagreb, Zagreb, Croatia; ^5^Department of Pathology, University Hospital Center Zagreb, Zagreb, Croatia

**Keywords:** gastrointestinal lesion, gastrointestinal bleeding, colon rupture, chronic kidney disease, sevelamer carbonate, case report

## Abstract

Chronic kidney disease (CKD) is a very common chronic non-communicable disease. Phosphate and calcium metabolism disorders are one of the most common features of CKD. Sevelamer carbonate is the most widely used non-calcium phosphate binder. Gastrointestinal (GI) injury associated with sevelamer use is a documented adverse effect but is underrecognized as a cause of gastrointestinal symptoms in patients with CKD. We report a case of a 74-year-old woman taking low-dose sevelamer with serious gastrointestinal adverse effects causing colon rupture and severe gastrointestinal bleeding.

## 1. Introduction

Sevelamer is an anion exchange resin used to lower hyperphosphatemia in CKD. Originally approved in 1998 as sevelamer hydrochloride, it has been largely replaced by sevelamer carbonate. Although both medications have similar efficacy, sevelamer carbonate has a lower risk of metabolic acidosis (1). Treatment of hyperphosphatemia is crucial in CKD patients, given its well-known association with vascular and endothelial damage, as well as increased mortality (2–4). Sevelamer is a calcium-free phosphate binder, thus avoiding the positive calcium balance induced by calcium-based binders, which is associated with increased mortality in CKD patients (5). It has also been suggested that sevelamer has beneficial pleiotropic effects (6). Sevelamer is a non-absorbable resin able to bind the phosphate in the gastrointestinal tract. Although side effects reported in patients receiving sevelamer include nausea, diarrhea, vomiting, dyspepsia, abdominal pain, flatulence, and constipation, there is little research to determine the mechanism of gastrointestinal symptoms (7). Sevelamer can also cause gastrointestinal tract motility disorders, which is mostly attributable to sevelamer hydrochloride (7).

Sevelamer-associated gastrointestinal injury presents a diagnostic challenge and is probably underrecognized cause of gastrointestinal symptoms in patients with CKD. Sevelamer has been shown to have mucosal deposits throughout the gastrointestinal tract with great variability in associated symptoms. However, the most common clinical presentation of sevelamer-induced GI lesions is GI bleeding, followed by acute abdomen and GI discomfort (8). Diabetics seem to be more prone to develop sevelamer-associated GI lesions (8). Mucosal ulceration including wall perforation and post-inflammatory stricture formation are among the serious complications (10–16). There is also one report describing sevelamer induce colitis presenting as pseudotumor (11). Some patients with sevelamer-induced GI lesions had previous GI diseases (dyspepsia, peptic ulcer, diabetic gastroparesis, ulcerative colitis), GI infection caused by Clostridium difficile, or major abdominal surgery (8–16). Concomitant anticoagulant and/or antiplatelet therapy may also have influenced the clinical presentation (8–16). The average daily dose of sevelamer taken by the patients with GI lesions is 4.8 g (range 0.8–9.6 g), but the association between the sevelamer dosage and the severity of GI lesions was not clearly established (8). There is also no clear association between the form of the drug (powder or tablet) or anion content (carbonate or hydrochloride) and the quantity or severity of GI lesions (8). The summary of previous reports of gastrointestinal lesions induced by sevelamer is presented in Table 1.

**Table 1:** Summary of previous reports of GI lesions induced by sevelamer.

**Author/Year of publication**	**Number of cases**	**Age (years)/Gender**	**Preexisting GI disease**	**Sevelamer dosage (grams/day)**	**Sevelamer crystals in mucosa**
Swanson et al. (9)	Case 1 Case 2 Case 3 Case 4 Case 5 Case 6 Case 7	59/f 68/m 38/m 49/f 53/m 66/m 81/m	No No Yes No No Yes No	9.6 4.8 4.8 7.2 2.4 N/A 4.8	Yes Yes Yes Yes Yes Yes Yes
Chintamaneni et al. (14)	Case 1	61/ f	No	7.2	Yes
Okwara et al. (11)	Case 1	79/m	No	N/A	Yes
Kim et al. (13)	Case 1	17/f	Yes	2.4	Yes
Tieu et al. (12)	Case 1	74/f	Yes	4.8	Yes
Yamaguchi et al. (10)	Case 1	66/m	No	4.5	Yes
Yuste et al. (8)	Case 1 Case 2 Case 3	51/f 53/m 76/f	No Yes Yes	8.8 9 0.8	Yes Yes Yes
Uy et al. (16)	Case 1	33/m	Yes	7.2	Yes
Cockrell et al. (15)	Case 1	65/f	N/A	N/A	Yes

GI-gastrointestinal, f-female, m-male, N/A-not applicable.

Notable mucosal abnormalities found in patients with sevelamer-associated GI lesions include chronic mucosal damage, acute inflammation, inflammatory polyps, extensive ulceration, ischemia, and necrosis (9). Histological features of sevelamer crystals include irregularly arranged “fish-scale” crystals found within the GI mucosa with different colors during *in vitro* and *in vivo* studies (9). Patients with sevelamer deposits in the GI tract can also be asymptomatic (8).

As mentioned above, there is little information documenting serious gastrointestinal bleeding or colon rupture with sevelamer as a cause. Here we describe a case of sevelamer-induced various life-threatening gastrointestinal complications, namely bleeding, and pseudotumor, in a single patient on a low dose of sevelamer.

## 2. Case report

A 74-year-old woman with several comorbidities (arterial hypertension, diabetes mellitus, valvular heart disease, and chronic kidney disease) was admitted to the hospital due to lower gastrointestinal bleeding resulting in hemodynamic instability and shock.

The initial event of lower gastrointestinal bleeding was documented during the hospitalization 7 months earlier. At that point, the patient presented with rectorrhagia and worsening renal function. A multi-slice computed tomography scan scan revealed a thickened wall of the ascending colon and hepatic flexure with no other significant pathology. Lower endoscopy showed inflamed and friable mucosa of the entire circumference of the transverse colon, splenic flexure, and part of descending colon with normal rectal mucosa. Taking into account the typical endoscopic appearance, the patient was diagnosed with ischemic colitis. Her diabetes was well controlled with dietary measures. She had high C-reactive protein levels and was treated with antibiotics including ciprofloxacin and metronidazole which resulted in complete clinical and laboratory normalization (Table 2). Stool cultures were negative. Due to unspecific inflammation in the colon mesalazine was added (3 g/day). At discharge, the treatment with sevelamer carbonate was induced (1.6 g /day) due to worsening of the kidney function and hyperphosphatemia. Importantly, the patient was not taking sodium polystyrene sulfonate or any other cation exchange resin. Mesalazine therapy was stopped after 3 months since the patient had no GI symptoms.

**Table 2:** The laboratory results and clinical course of the patient.

	**January 2022 Hospitalization due to rectorrhagia**	**October 2022** **Hospitalization due to rectorrhagia**	**October 2022 Hospitalization due to severe rectorrhagia, hypotension**
	Therapy (daily dosage): propranolol 40 mg Furosemide 40 mg Amlodipine 5 mg Perindopril 8 mg Vitamin D supplement Ciprofloxacin 250 mg metronidazole 1200 mg Mesalazine 3 g (3 months course) Sevelamer carbonate 1600mg (beginning of treatment- at discharge)	Therapy (daily dosage): propranolol 40 mg Furosemide 40 mg Vitamin D supplement Sevelamer carbonate 1,600 mg Meropenem 500 mg i.v. Mesalazine 3 g (reintroduced) Prednisone 40 mg	Therapy (daily dosage): propranolol 40 mg Furosemide 40 mg, vitamin D Sevelamer carbonate 1,600 mg (discontinued) Meropenem 500 mg i.v. Mesalazine 3 g Prednisone 40 mg (discontinued) Fluid replacement Blood transfusions Ciprofloxacin 400 mg Metronizdol 1500 mg Total colectomy, formation of terminal ileostomy
Hemoglobin (RR 119–157 g/l)	92	106	70
Hematocrit (RR 35-47 %)	28	33	22
Leucocytes (RR 3.4–9.7x10E9/l)	7.7	17.8	32.8
Creatinine (49–90 μmol/l)	466	290	207
GFR (RR> 60 ml/min/1.73m2)	< 15	< 15	20
Potassium (RR 3.7–5.0 mmol/l)	5.2	5.6	4.2
Calcium (RR 2.14–2.53 mmol/l)	1.93	2.09	N/A
Phosphorus (RR 0.79–1.42 mmol/l)	2.04	1.00	N/A
C-reactive protein (RR < 5 mg/l)	74.9–17.5–12.1	120.9–34.9	11
Stool cultures	negative	negative	N/A
Endoscopy	Multiple lesions in descendent and transverse colon	Multiple ulcerations in transverse colon	The lumen of colon filled with fresh blood; it was not possible to visualize the bleeding site
Pathohistological finding	Non-specific inflammation Ischemic colitis	N/A	Severe mucosal injury, acute inflammation within the bowel wall, sevelamer crystal within the luminal debris

RR, reference range, N/A, not applicable.

**Figure 1:**
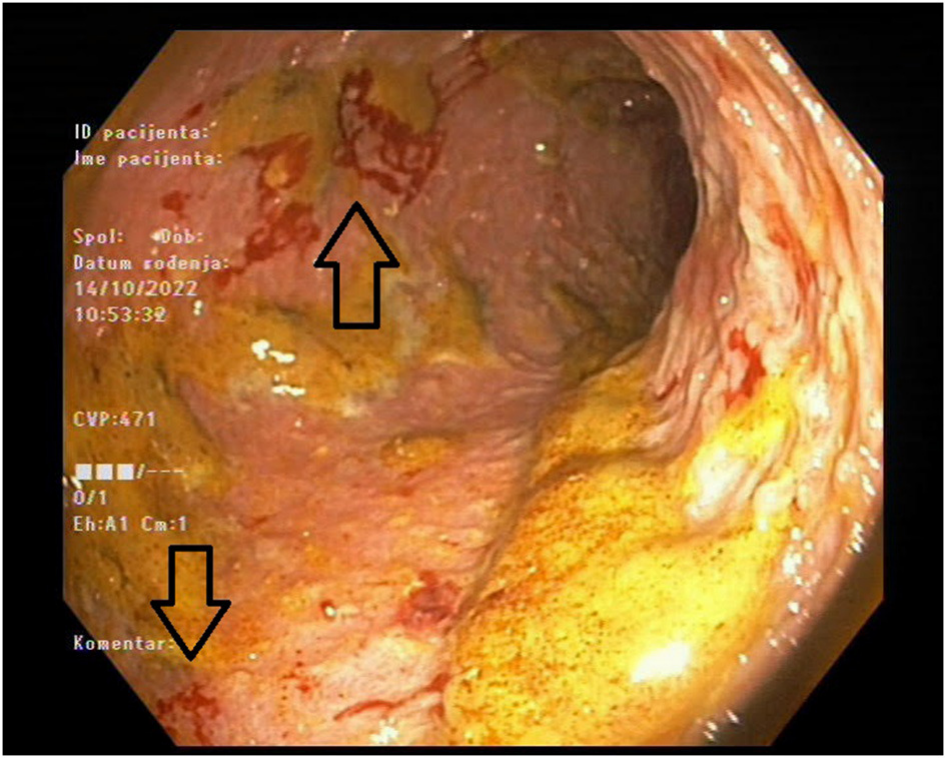
Colon endoscopy showing diffusely inflamed and ulcerated mucosa of transverse colon.

The patient was readmitted after 7 months and presented with diarrhea and abdominal pain. Her laboratory results showed again significantly increased inflammatory markers, but with negative stool cultures. Her kidney function was stable (Table 2). Endoscopy was performed and revealed a large ulceration in the transverse colon encompassing the whole circumference of the colon (Figure 1). A multi-slice computed tomography scan showed a significantly thickened wall of the cecum, ascending and transversal colon.

The patient was treated empirically with meropenem alongside other supportive treatments. Due to the presence of inflammatory changes in the colon, inflammatory bowel disease was suspected and, prednisone was started with rapid clinical and laboratory improvement. The patient was discharged after 10 days of hospitalization with normal bowel movements and no other symptoms. Two days after discharge, she was readmitted due to severe lower gastrointestinal bleeding with hemorrhagic shock. Despite the intensive treatment measures the patient remained unstable, and given the impossibility of definitive endoscopic hemostasis with hemodynamic instability patient was referred to urgent surgery.

**Figure 2:**
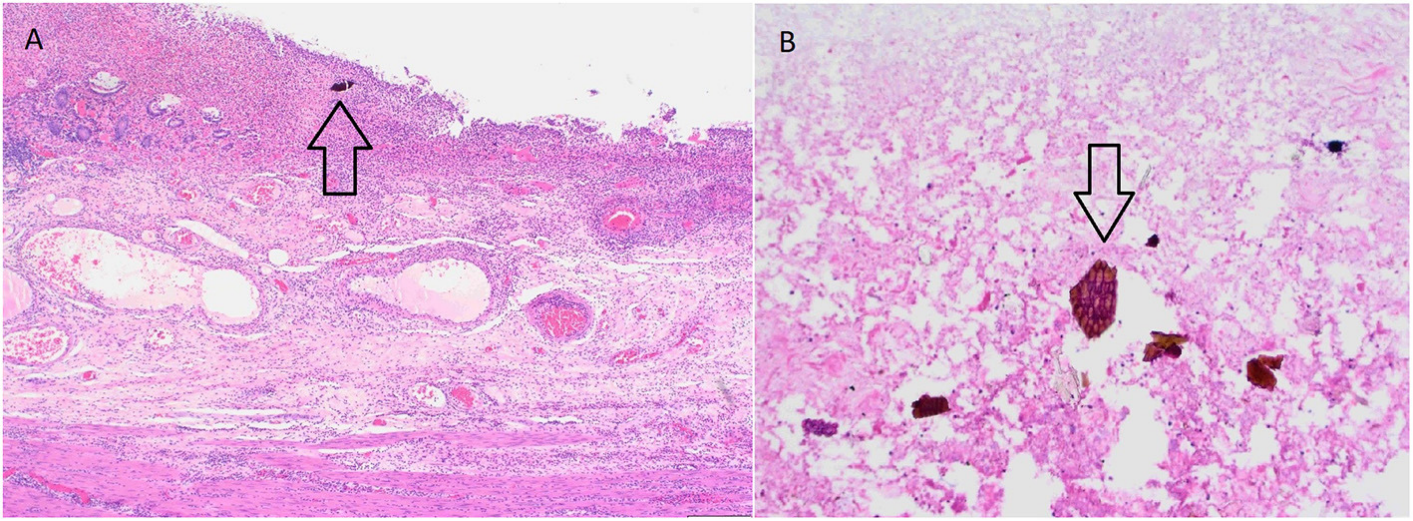
**(A)** Severe mucosal injury with acute inflammation within the bowel wall. Note the sevelamer crystal within the luminal debris. (H&E, 40x). **(B)** Sevelamer crystal within the luminal debris—characteristic fish scale appearance with orange and pinkish hue (H&E, 200x).

Intraoperatively, the colon was described as malignantly altered, with the tumor-changed central part of the transverse colon, and the retained perforation. The total colectomy and terminal ileostomy were performed. The histopathological finding of the colon described severe mucosal injury with acute inflammation and visible characteristic “fish-scale” crystals of sevelamer within the luminal debris, accompanied by acute inflammation and focal necrosis of the bowel wall, with no signs of dysplasia or malignant alteration (Figures 2A, B). The conclusion of these findings was that the necrosis of the colonic wall was caused by sevelamer, and the drug was discontinued. Postsurgical recovery was complicated by an episode of cardiac decompensation which resolved with optimized diuretic treatment. The patient was discharged after 8 days of hospitalization in stable clinical condition.

## 3. Discussion

Sevelamer is an anion-exchange resin designed for the treatment of hyperphosphatemia in patients with chronic renal disease. Its structure is made of a non-absorbed hydrogel with ammonia on the hydrochloride or the carbonate. Due to the acidic content of the stomach, the polymer dissociates from its anion and consequently is available to bind phosphate within the intestine (17). Sevelamer also causes dehydration in the intestinal tract, resulting in the formation of hard stool (7).

Despite the side effects reported in patients receiving sevelamer, e.g., nausea, diarrhea, vomiting, dyspepsia, abdominal pain, flatulence, and constipation, there is little research to determine the direct mechanism of sevelamer on the gastrointestinal tract (18–20). Post-marketing data show rare cases of ileus and intestinal obstruction, attributable mainly to sevelamer hydrochloride, but the frequency of severe intestinal adverse effects is unknown (7). The direct effect of hard stool and constipation following sevelamer administration may trigger perforation, but given the lack of investigation the potential cause remains unclear. Moreover, in patients undergoing hemodialysis, hypotension episodes and hypovolemia may cause mesenteric ischemia and intestinal necrosis, making the mucosa more vulnerable to other causes of injury (9, 10). Swanson et al. described the histopathology of sevelamer crystals in the GI tract, and their association with mucosal abnormalities which can be found in the esophagus, small bowel, and colon (9).

It has been reported that patients on hemodialysis have a high risk of colonic perforation even without sevelamer administration, and there were no significant changes in the incidence of bowel perforation after the approval of sevelamer (21, 22).

In our case, the patient had the serious adverse effects of sevelamer therapy presenting with life-threatening GI hemorrhage. Although our patient was diagnosed with ischemic colitis, she responded well to mesalazine therapy and had no GI symptoms. The sevelamer was induced after the first hospitalization when her GI symptoms resolved. Thus, we cannot conclude with certainty that our patient had an underlying GI disease, contrary to theother reports in the literature (8–16). On the other hand, our patient is diabetic, which makes her more prone to sevelamer-induced GI lesions (8). Our patient was treated with a relatively low dose of sevelamer (1.6 g/day), and the duration of therapy was only seven months. However, previous reports did not establish the association between the sevelamer dosage and the severity of GI lesions, which occurred also in patients taking 800 mg sevelamer daily (8). Moreover, our patient showed characteristic histopathological findings of sevelamer crystals in lesion biopsies and direct deposition of sevelamer crystals in areas of ulceration and pseudotumor formation was found. In conclusion, we believe that sevelamer use is the most likely etiology of pseudotumor formation and restrained rupture of the transverse colon in our patient. Sevelamer-induced GI lesions should always be considered in CKD patients treated with sevelamer who present with GI symptoms, especially lower GI bleeding, regardless of the sevelamer dosage. Sevelamer therapy should be prescribed with caution in patients with a history of major abdominal surgery, chronic GI disease, and diabetes given the high risk of serious GI complications in this patient population.

## 4. Patient perspective

Our patient was hospitalized twice in a short period of time due to abdominal pain and bloody stools. Her appetite was poor and she was feeling ill, but responded well to treatment. A few days after being discharged, she was again hospitalized due to bloody stools and abdominal pain. She felt very weak. After the surgery, she had breathing difficulties, which resolved after therapy. She was discharged from the hospital and is currently in-home care.

## Data availability statement

The original contributions presented in the study are included in the article/supplementary material, further inquiries can be directed to the corresponding author.

## Ethics statement

Written informed consent was obtained from the individual(s) for the publication of any potentially identifiable images or data included in this article.

## Author contributions

MF, MJ, MB, DG, and IR: substantial contribution to the conception of the work, interpretation of findings, drafting the work, and final approval of the report. ZM: analysis of kidney biopsy material, interpretation of findings, critical revising of data for the work, and final approval of the report. EI: substantial contribution to the conception of the work, interpretation of data, critical revising of the work, and final approval of the report. BJ, IV, and ZK: interpretation of data, critical revising of the work, and final approval of the report. All authors contributed to the article and approved the submitted version.
